# Gut microbial characteristics of adult patients with allergy rhinitis

**DOI:** 10.1186/s12934-020-01430-0

**Published:** 2020-09-01

**Authors:** Libing Zhu, Feng Xu, Wenrong Wan, Bin Yu, Lin Tang, Yiming Yang, Yanling Du, Zhangran Chen, Hongzhu Xu

**Affiliations:** 1 Department of Traditional Chinese Medicine, School of Medicine, Xiamen University, Xiamen, China; 2 Internal Medicine Department of Traditional Chinese Medicine, Xiamen Hospital of Traditional Chinese Medicine, Xiamen, China; 3 Department of Otorhinolaryngology, Xiamen Hospital of Traditional Chinese Medicine, Xiamen, China; 4 Department of Pediatrics, Xiamen Hospital of Traditional Chinese Medicine, Xiamen, China; 5 Department of Acupuncture and Tuina, Fujian University of Traditional Chinese Medicine, Fuzhou, China; 6 Department of Digestive Diseases, School of Medicine, Xiamen University, Xiamen, China; 7 Department of Gastroenterology, Zhongshan Hospital, Xiamen University, Xiamen, China; 8 Institute for Microbial Ecology, School of Medicine, Xiamen University, Xiamen, China

**Keywords:** Allergy rhinitis, Gut dysbiosis, Total nasal symptom score, Functional pathway

## Abstract

**Background:**

Although recent studies have indicated that intestinal microbiota dweller are involved in the pathogenesis of allergy rhinitis (AR), the influence of gut microbiota on AR adult has not been fully elucidated yet. Hence, we carried out this study to uncover the distinctive bacterial taxa that differentiate allergy rhinitis patients from healthy individuals. Feces samples from thirty three AR patients and thirty one healthy individuals were analyzed by 16S rRNA gene sequencing.

**Results:**

Results showed that the bacterial diversity in AR group was significantly higher than that of the non-AR group. Bacterial communities between AR and non-AR group were significantly differentiated as revealed by Principal coordinates analysis (PCoA) and the variation within non-AR were higher than that of the counterpart. Firmicutes, Fusobacteria, Actinobacteria, Cyanobacteria and Chloroflexi were the significantly differed phyla taxa and the top significantly distinguished bacterial genus included *Prevotella*_9, *Phascolarctobacterium*, *Roseburia*, *Megamonas*, *Alistipes*, *Lachnoclostridium* and *Fusobacterium*. The higher network complexity in AR group were dominated by taxa belonging to Firmicutes. The predicted function, alpha linolenic acid metabolism and bacterial invasion of epithelial cells pathway were higher in non-AR group while gonadotropin-releasing hormone (GnRH) signaling pathway, Fc γ-R mediated phagocytosis and endocytosis were higher in AR patients. Although the bacterial diversity between moderate and severe AR patients showed no significant difference, the significant correlation between featured genus and total nasal symptom score or rhinoconjunctivitis quality of life questionnaire, such as *Butyricicoccus* and *Eisenbergiella*, revealed the potential to intervene the AR status by means of gut microbiota.

**Conclusions:**

In conclusion, patients with allergy rhinitis had distinguished gut microbiota characteritics in comparison with healthy controls. The results suggest that gut microbiota might play crucial roles in influencing the course and different symptoms of AR.

*Trial registration* ChiCTR, ChiCTR1900028613. Registered 29 December 2019, https://www.chictr.org.cn/showproj.aspx?proj=47650.

## Background

Allergic rhinitis (AR) is one of the most globally common diseases and usually persists throughout life [1, 2]. AR is defined as immunoglobulin E (IgE)-mediated noninfective inflammatory disease of the nasal mucosa after allergen exposure and a variety of immune active cells and cytokines are involved [3]. The classic symptoms of AR include nasal congestion, nasal itching, rhinorrhea and sneezing [4, 5]. Conservatively, more than 500 million people suffer from allergic rhinitis around the world [1]. AR impose a significantly adverse effect on the quality of life [1] and increase a risk for bronchial asthma, rhinosinusitis, nasal polyps, otitis media and allergic conjunctivitis [6, 7].

The mainstay conventional treatment for AR includes allergen avoidance, pharmacotherapy, immunotherapy and patient education [1, 4]. Although the recommended first-line therapy (nasal glucocorticosteroids and leukotriene receptor antagonists) [1, 4, 8] may be effective in reducing the symptoms of AR, the concomitant adverse effects during long-term use make them awedness. For example, second-generation H1 antihistamines which are frequently used for AR have limited efficacy in treating nasal congestion, commonly induce headache and drowsiness [9, 10]. These first-line drugs can be successfully used to control AR. However, once these medications are terminated, the majority of AR patients will reappear the symptoms of AR within a brief period. Thus, these medications do not appear to exert a long-term effect on the baseline Total Nasal Symptom Score (TNSS).

Recently, the role of probiotics in nutritional interventions have been investigated regarding to their beneficial effects on AR in improving patients’ quality of life and reducing medication use [11, 12]. The beneficial roles of probiotics in allergic diseases have been investigated with increasing interest in animal models and human clinical trials [13–15], which indicate their significant influence on the gut microbiota composition and host immune system restoration [16, 17]. For example, *Lactobacillus casei* Shirota strain exerted effect on the immune system and allergic symptoms [18, 19], while *Lactobacillus plantarum* showed the ability to inhibit allergic cytokines and *Bifidobacterium longum* BB536 strain alleviated symptoms of seasonal AR [20–22]. Hence, considering the unsatisfactory results of long-term AR treatment, a better understanding of relationship between gut microbiota and AR disease, and the further potential regulation mechanism of gut microbiota for AR is necessary.

Consequently, in this study, we compared the intestinal microbiota characteristics between 31 non-AR and 33 AR patients through high-throughput 16S rRNA gene sequencing. The present study was to: (1) compare the composition and functional features of gut microbiota between AR and non-AR individuals; (2) identify the specific gut microbiota which was closely related to the severity of AR as well as the potential pathway of gut microbiota interacting with the host.

## Methods and materials

### Clinical trial number

The ethics committee of Xiamen hospital of Traditional Chinese Medicine approved this study (Permit Number ID: 2019-K020-01) and later it was registered in Chinese Clinical Trial Registry (ChiCTR1900028613). All healthy individuals and patients made agreement on the informed consent, and the study protocol complied with the ethical guidelines of the Declaration of Helsinki [23].

### Non-AR Individuals

Thirty one non-AR populations were enrolled from December 2017 to May 2018 at Zhongshan Hospital, affiliated with Xiamen University (Xiamen, China). The inclusion criteria were as follows: (1) no allergic history and no family history of allergic diseases; (2) sausage or snake shaped stool; and (3) voluntary participation in this study. The exclusion criteria of non-AR populations were based on previously reported [24] and individuals with allergy disease, such as AR, asthma, eczema and urticarial were further excluded.

### Study subjects and sample collection

Thirty three AR patients were recruited at Xiamen hospital of Traditional Chinese Medicine (Xiamen, China) from December 2019 to March 2020. Patients were recruited with the following inclusion criteria: (1) age of 18–65 years; (2) meet the international diagnostic standard of AR (Allergic Rhinitis and its impact on Asthma, ARIA) [25]: (a) the symptoms of sneezing, nasal congestion, nasal itching and rhinorrhea appear two or more, and the symptoms last or accumulate for more than 1hour every day; (b) pale and edematous nasal mucosa, nasal water like secretion; (c) at least one allergen skin prick test (SPT) and/or serum specific IgE was positive; (3) greater than or equal to 4 days every week, and four or more consecutive weeks; and (4) voluntary participation in this study.

AR patients who meet one of the following criteria were excluded: (1) upper respiratory tract infection, chronic obstructive pulmonary disease, emphysema, bronchiectasis, tuberculosis, pneumonia, bronchial asthma, secretory otitis media; (2) postural hypotension, organic heart disease or clinically significant ECG changes; (3) patients with thyroid disease, diabetes mellitus, hypoglycemia, cardiovascular and cerebrovascular disease, prostatic hypertrophy, glaucoma, epilepsy history, liver and kidney dysfunction, hematopoietic dysfunction, gastrointestinal diseases, malignant tumors; (4) patients had a history of mental and neurological diseases; (5) patients who had received the following treatment before the experiment: immunotherapy within 12 months, long-acting glucocorticoid injection therapy within 3 months, asmidazole within 6 weeks, glucocorticoids for systemic or local use within 2 weeks (inhalation or nasal spray), sodium cromoglycerin or naidomide within 1 month, long-acting antihistamines within 2 weeks, short-acting antihistamines within 1 week, ketotifen, tricyclic antidepressants, macrolides and antifungal drugs within 2 weeks, systemic or local nasal decongestants within 24 h, using anti-histamine drugs, anticholinergics and antiarrhythmic drugs currently, using barbiturates, antispasmodics, phentolamine and digitalis drugs currently; or (6) abnormal physical examination results with clinical significance before admission, which may affect the achievement of the purpose of this study or hinder the realization of compliance with the research procedures according to the judgment of the researchers.

### TNSS and Rhinoconjunctivitis Quality of Life Questionnaire (RQLQ)

TNSS and RQLQ were performed for AR patients to assess the severity of AR and the impact on patients’ quality of life caused by AR during the enrollment. Nasal symptoms were assessed using the TNSS score [26]. RQLQ consist of 28 items in seven domains and each dimension was scored separately, and the cumulative total score was the total RQLQ score [27, 28].

### Lab work and the bioinformatics analysis

The procedure for DNA extraction of fecal samples (approximately 0.25 g) from non-AR healthy individuals and AR patients, the purity and concentration of the isolated DNAs, the 16S rRNA V3–V4 hypervariable region amplification for the library construction and paired-end sequencing were based on previous procedure [24]. The bioinformatics analysis procedure for raw reads were according to the previously described [24, 29].

### Statistical analysis

Bacterial alpha diversity indices, bacterial community ordination based on Bray-Curis distance, PERMANOVA and anosim analysis were based on the R package vegan (version 2.5.5) [30]. The group difference comparison were conducted by ANOVA tests. The significantly distinguished taxa and predicted pathway by PICRUSt [31] were screened by comparison between AR and non-AR individuals by Wilcox test and LEfSe analysis. The sankey plots showing the genus features for AR and non-AR group were made by networkD3 packages (version 0.4). Pearson’s correlations between the abundances of differential genus taxa and pathway were computed by the R package stats (version 3.6.0) and package pheatmap (version 1.0.12) were used to conduct the correlation heatmap. Networks were visualized using the Gephi 0.9.1 [32]. These analyses were performed using R3.5.1 [33].

## Results

### The clinical characteristics of participant

In this study, we used feces collected from 33 AR patients and 31 non-AR individuals (Additional file 1: Table S1). There was no significantly difference in age, body mass index (BMI) between AR and non-AR group (AR adults between the ages of 19 and 56 years; Non-AR between the ages of 18 and 55 years). SPT (Dust mite drops) was positive in all AR patients. Among AR individuals, the TNSS was 7.33 ± 2.41 (Nasal obstruction: 1.70 ± 0.92, rhinorrhea: 1.91 ± 1.01, nasal itching: 1.67 ± 0.92, sneezing: 2.06 ± 0.79.) and the RQLQ was 68.73 ± 35.76 (Sleep problems: 5.91 ± 4.78, non-eye/nasal symptoms: 16.76 ± 10.70, practical problems: 9.52 ± 5.19, eye symptoms: 6.70 ± 5.88, nasal symptoms: 12.15 ± 5.72, activity limitation: 9.70 ± 4.66, emotions problems: 8.00 ± 5.77) (Table.1).

**Table 1:** Clinical characteristics of AR and non-AR subjects

Group	Adults	
	Non-AR	AR
Subjects (n)	31	33
Age (years)^#^	32.06 ± 9.26	31.79 ± 9.91
Gender		
Male (%)	18	11
Female (%)	13	22
BMI (kg/m^2^) ^#^	21.85 ± 2.57	21.86 ± 3.16
SPT (%)	0	100
TNSS	0	7.33 ± 2.41
Nasal obstruction	0	1.70 ± 0.92
Rhinorrhea	0	1.91 ± 1.01
Nasal itching	0	1.67 ± 0.92
Sneezing	0	2.06 ± 0.79
RQLQ	NA	68.73 ± 35.76
Sleep problems	NA	5.91 ± 4.78
Non-eye/nasal symptoms	NA	16.76 ± 10.70
Practical problems	NA	9.52 ± 5.19
Eye symptoms	NA	6.70 ± 5.88
Nasal symptoms	NA	12.15 ± 5.72
Activity limitation	NA	9.70 ± 4.66
Emotions problems	NA	8.00 ± 5.77

^#^means *p* > 0.05, no significant effect. NA: Not available. SPT, skin prick test, TNSS, Total Nasal Symptom Score. RQLQ, Rhinoconjunctivitis Quality of Life Questionnaire

### Comparison of bacterial diversity between AR and non-AR individuals

**Fig. 1:**
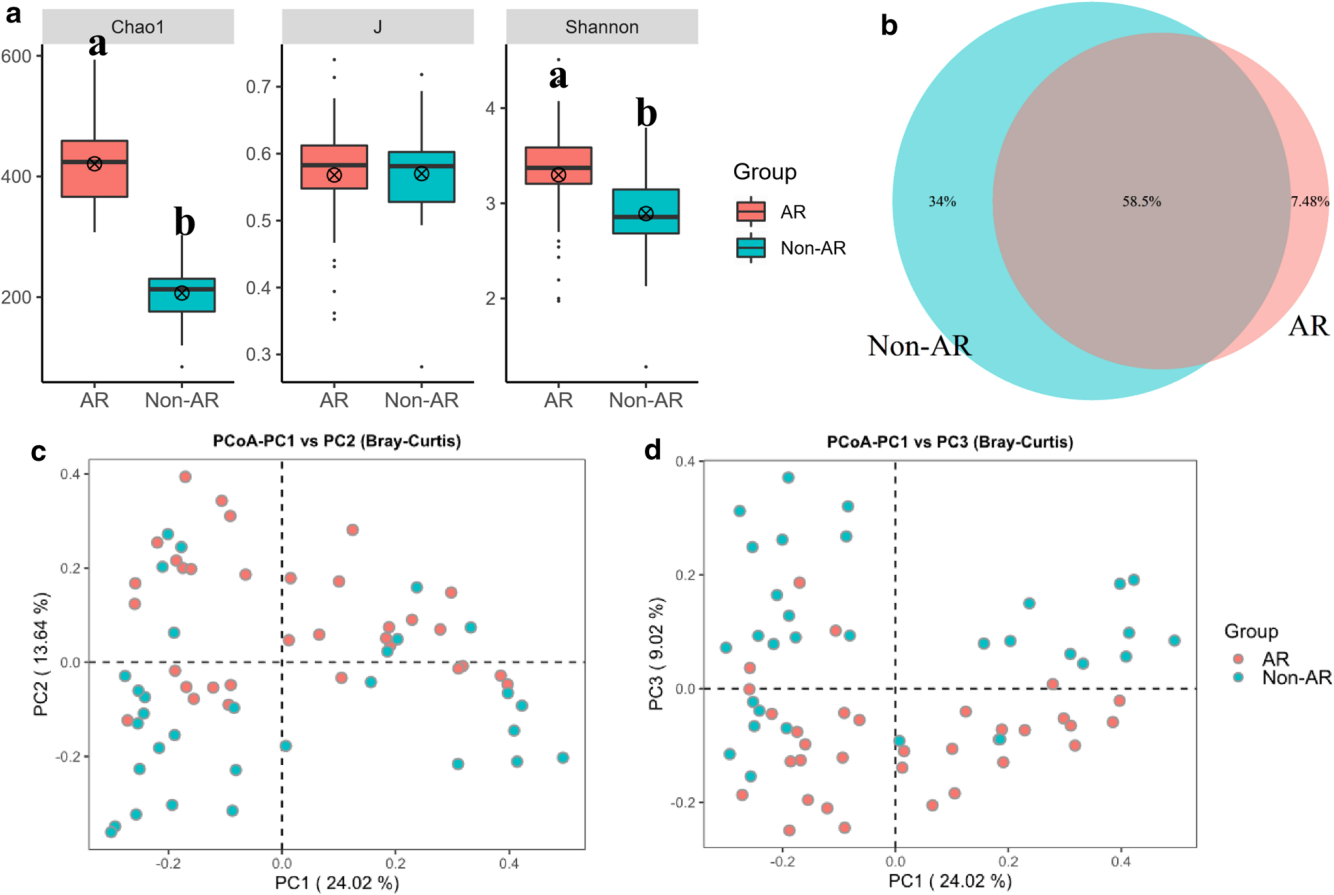
Comparisons of bacterial diversity between AR and non-AR patients. **a** The bacterial α diversity indexes comparison including Chao1, J and Shannon. Letters indicate the ANOVA groupings. **b** Differences in bacterial community structures between samples from AR and non-AR

A total of 5,875,233 raw reads (2,199,830 reads for AR patients; 3,675,403 reads for non-AR individuals), average of 91,800 OTU sequence/sample and an average 316 OTUs were obtained from 64 samples (Additional file 1: Table S2). The saturated rarefaction curves based on Chao1 and observed species indicated the high quality of sequence for all samples and the score of AR samples tended to be higher than the counterpart (Additional file 2: Figure S1). Ten iterations were made to obtain the alpha diversity metrics. There were significant differences between AR and non-AR individuals in diversity indices Chao1 and Shannon (*p* < 0.05) while not for J indices (*p* > 0.05) (Fig. 1a). For example, the average observe, Chao1 and ACE index from the AR patients (330,421,418) were twice times more than that of non-AR individuals (161,207,202) while the Simpson (0.87) and J indices (0.57) were similar (Additional file 1: Table S3). Shared “universal” OTUs (found in samples from all AR and non-AR patients) accounted for 58.5% of the total. Non-AR individuals contained more exclusive OTUs than that of AR patients (Fig. 1b).

Significant differences in microbial community structure between AR and the non-AR group were observed based on ANOSIM analyses (*p* < 0.001) (Additional file 2: Figure S2A). From the PCoA plot that were based on the Bray–Curtis distance, the microbiota was separated in the first axis (Fig. 1c, d) and the non-AR group tended to be more dispersed than AR patients which could be further verified by distance variation analysis (Additional file 2: Figure S2B)**,** the Bray–Curtis dissimilarity within non-AR individuals were significantly higher than that of within AR group.

### Comparison of bacterial community between AR and non-AR individuals

**Fig. 2:**
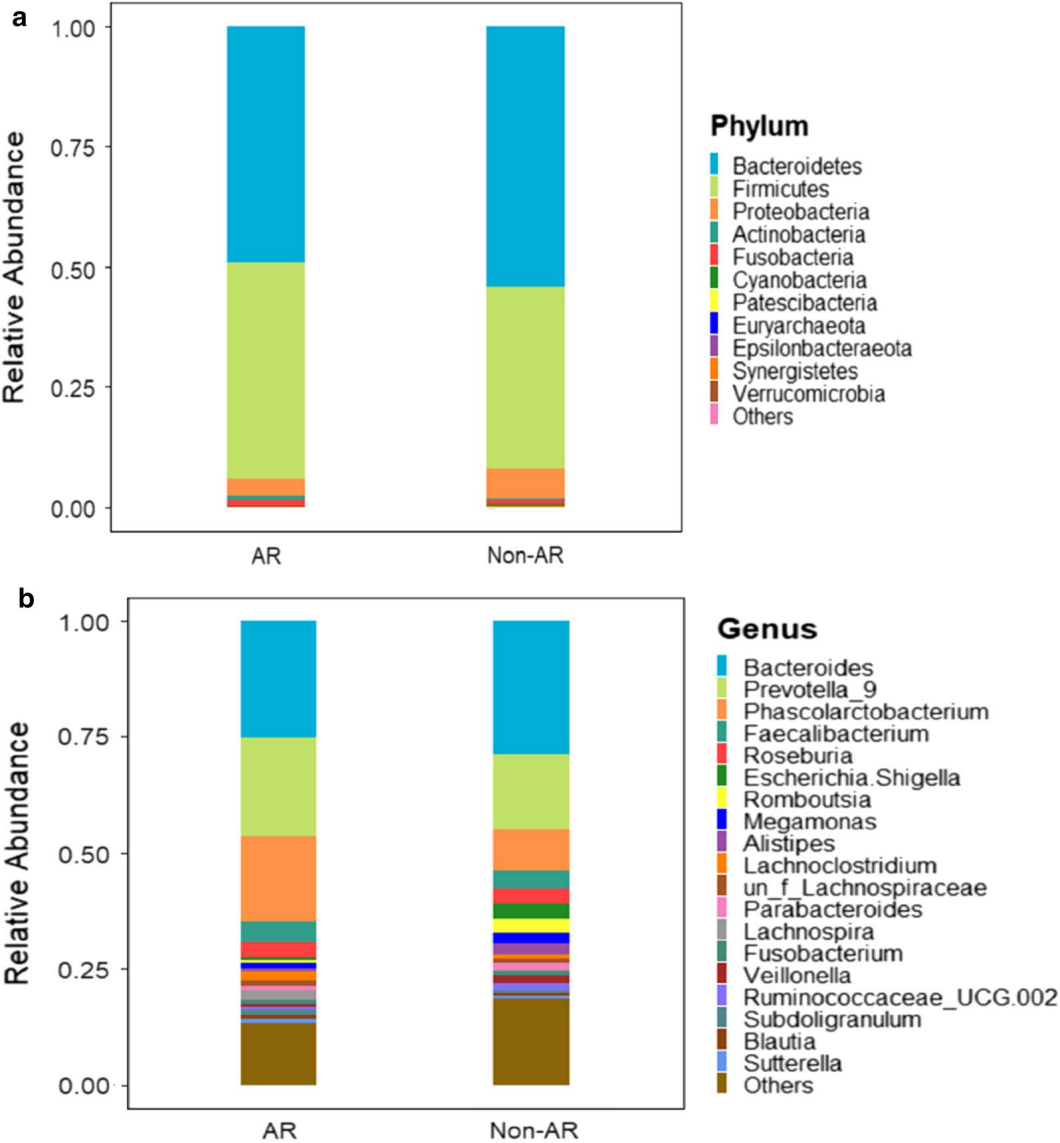
The bacterial structure comparison between AR and non-AR in phylum (**a**) and genus (**b**) level. Top 20 genus according to the relative abundance were included

**Fig. 3:**
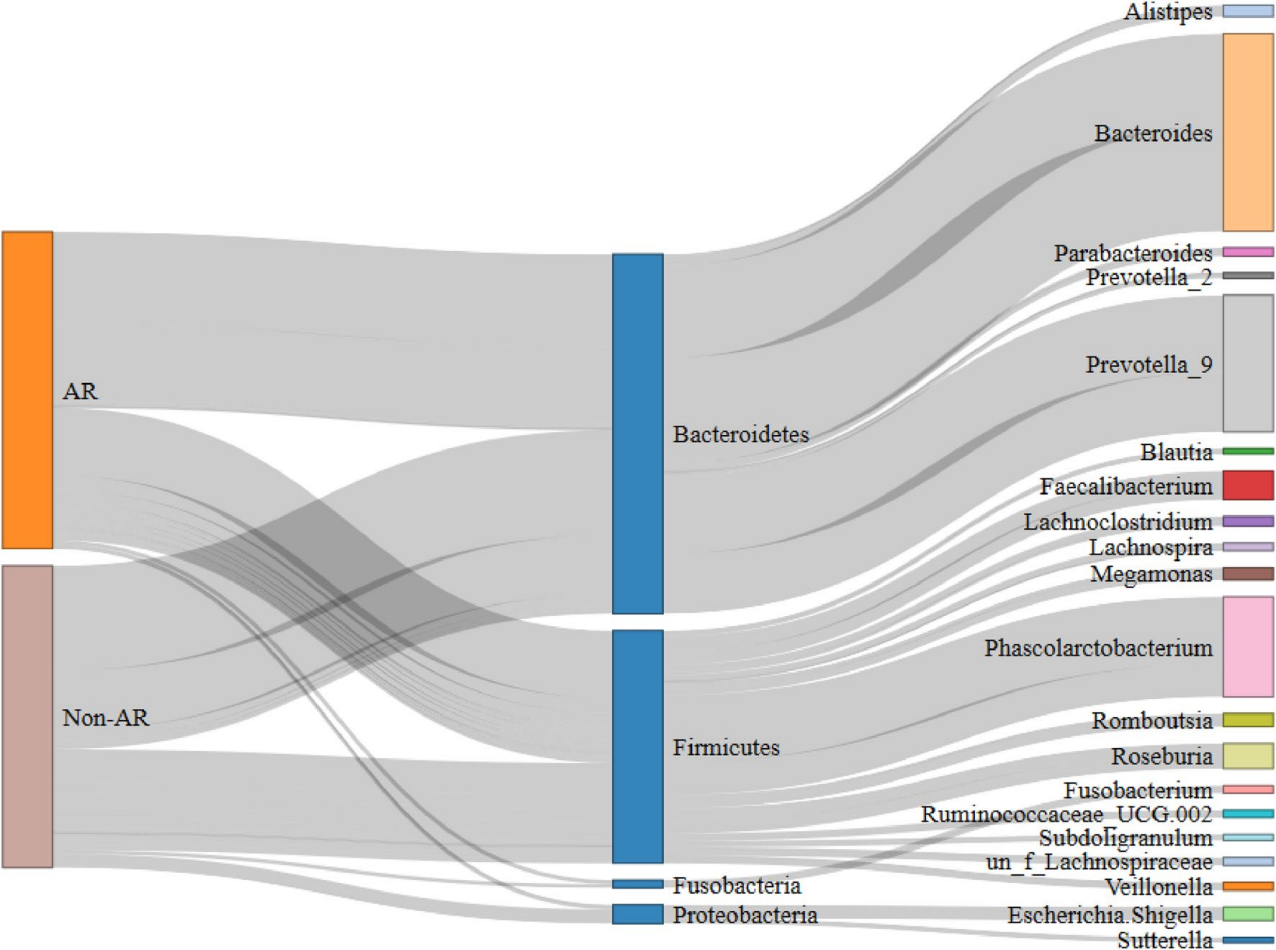
Sankey analysis of all non-AR and AR patients

Sixteen bacterial phyla were detected in total, Thereinto, Bacteroidetes, Firmicutes, Proteobacteria, Fusobacteria were the dominated taxa (occupying approximately 99.0%). In AR group, the average relative abundance of Bacteroidetes (49.27%) and Proteobacteria (3.61%) were lower than that in non-AR individuals (54.32%, 6.25% separately) while Chloroflexi and Spirochaetes were the unique taxa which were absent in non-AR parts (Additional file 1: Tables S4 and S5). Non-AR group had lower abundance of Firmicutes and Fusobacteria, but had the unique phyla Lentisphaerae (Fig. 2a). Two hundred genus were observed in total and *Bacteroides*, *Prevotella*_9, *Phascolarctobacterium*, *Faecalibacterium*, *Roseburia*, *Escherichia, Shigella*, *Romboutsia*, *Megamonas*, *Alistipes*, *Lachnoclostridium* were the top 10 genus which added up to 75.20% (Additional file 1: Table S6). Compared with non-AR group, AR patients displayed higher abundance of *Prevotella*_9, *Phascolarctobacterium*, *Faecalibacterium*, *Parabacteroides* while lesser in *Bacteroides*, *Megamonas*, *Romboutsia* and *Veillonella* (Fig. 2b and 3).

**Fig. 4:**
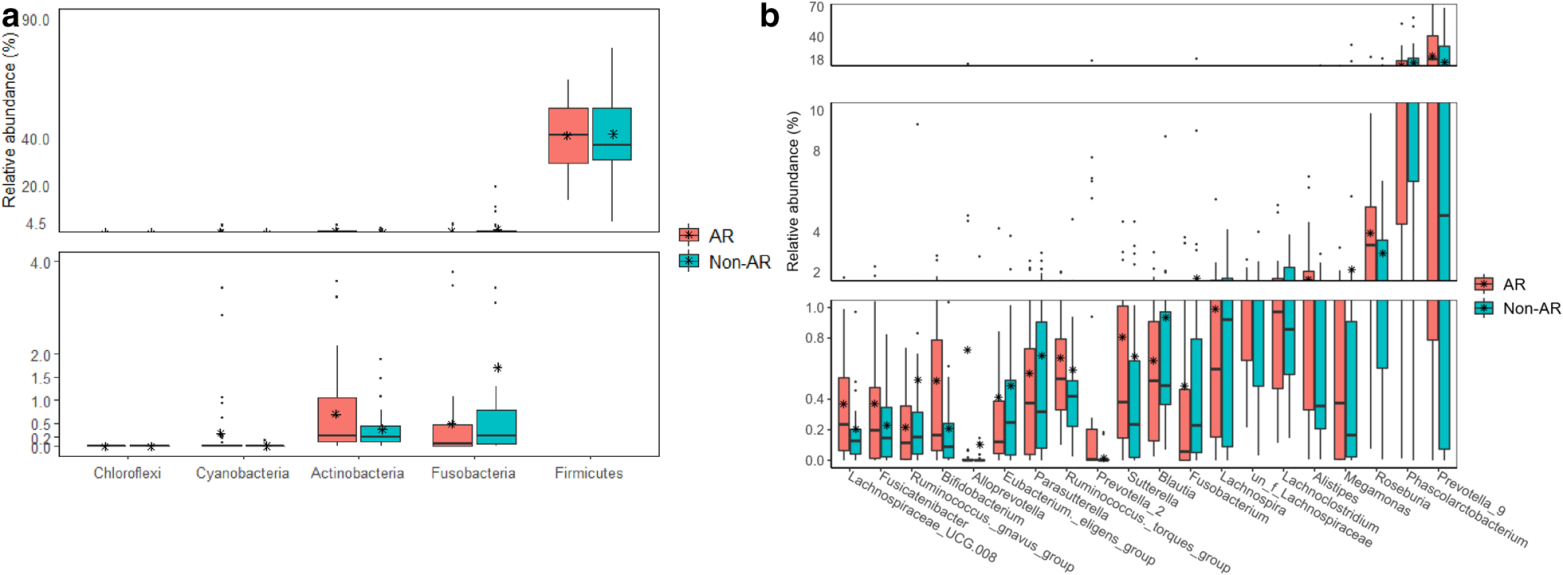
The relative abundance of distinguished bacterial taxa between non-AR and AR patients. **a** Differences in relative abundances of distinguished phyla screened by wilcox test. **b** Differences in relative abundances of top 20 distinguished genus screened by wilcox test (*p* < 0.05) and |logFC|> 1

Furthermore, the wilcox test were made to compare the significantly distinguished phyla and genus between AR and non-AR group. Firmicutes, Fusobacteria, Actinobacteria, Cyanobacteria and Chloroflexi were the five differential phyla (Fig. 4a). There were 76 significantly different genus and *Prevotella*_9, *Phascolarctobacterium*, *Roseburia*, *Megamonas*, *Alistipes* and *Lachnoclostridium* were the top ranked distinguished genus (Table2, Additional file 1: Table S7, Fig. 4b).

**Table 2:** Top 20 distinguished genus between AR patients and non-AR healthy individuals

Genus	AR(Average(Min–Max))	Non-AR(Average(Min–Max))
*Prevotella*_9	0.211(0.004–0.779)	0.163(0–0.548)
*Phascolarctobacterium*	0.185(0.038–0.593)	0.088(0–0.304)
*Roseburia*	0.035(0.009–0.126)	0.034(0–0.209)
*Megamonas*	0.012(0–0.120)	0.022(0–0.328)
*Alistipes*	0.006(0.001–0.053)	0.026(0–0.123)
*Lachnoclostridium*	0.019(0.004–0.053)	0.008(0.001–0.029)
un_f_Lachnospiraceae	0.013(0.004–0.026)	0.009(0–0.040)
*Lachnospira*	0.019(0.006–0.056)	0.003(0–0.013)
*Fusobacterium*	0.011(0–0.193)	0.010(0–0.107)
*Blautia*	0.009(0.002–0.022)	0.007(0–0.087)
*Sutterella*	0.009(0–0.045)	0.006(0–0.030)
*Prevotella*_2	0.003(0–0.067)	0.012(0–0.174)
*Ruminococcus*._torques_group	0.004(0.001–0.016)	0.008(0–0.046)
*Parasutterella*	0.009(0.001–0.030)	0.003(0–0.015)
*Eubacterium*._eligens_group	0.007(0.001–0.042)	0.001(0–0.022)
*Alloprevotella*	0.001(0–0.028)	0.008(0–0.142)
*Bifidobacterium*	0.005(0.001–0.028)	0.002(0–0.018)
*Ruminococcus*.gnavus_group	0.003(0–0.016)	0.004(0–0.093)
*Fusicatenibacter*	0.005(0.001–0.023)	0(0–0.002)
Lachnospiraceae_UCG.008	0.004(0–0.018)	0.002(0–0.010)

These 20 genus are screened out based on wilcox.test (*p* < 0.05) and |log(Fold change)|> 1 between the two groups

### Higher complicated network pattern in AR patients

**Fig. 5:**
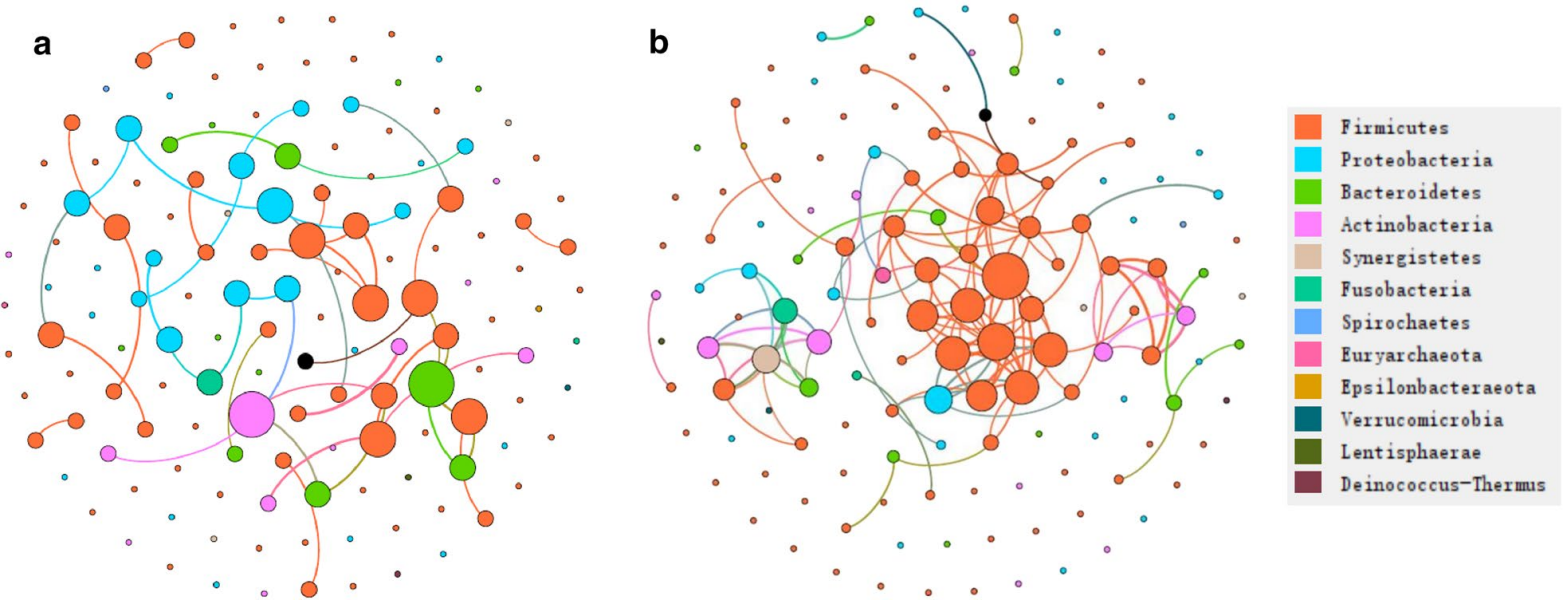
The network analysis between bacterial taxa for non-AR (**a**) and AR (**b**) group. Different node color denotes varied phyla taxa and the weighted node size were based on the relative abundance. The weighted edges indicates the correlation coefficient

For the non-AR group, whose modularity, nodes and edges were 0.86, 142 and 45 separately, Actinobacteria, Bacteroidetes and Proteobacteria might play more important roles than that of in AR, and *Slackia*, *Raoultibacter* that are within Actinobacteria, were highly correlated with *Eubacterium brachy*, DTU089 within Firmicutes. In the AR group, the clustered nodes mainly included *Anaerofilum*, *Olsenella*, *Pseudobutyrivibrio* within Firmicutes, *Olsenella* within Actinobacteria (Additional file 1: Table S8). The edges, nodes, modularity for AR group were 125,142 and 0.67 separately. Hence, AR tended to have more complicated network topology (Fig. 5).

### Association between featured bacterial taxa and pathway

**Fig. 6:**
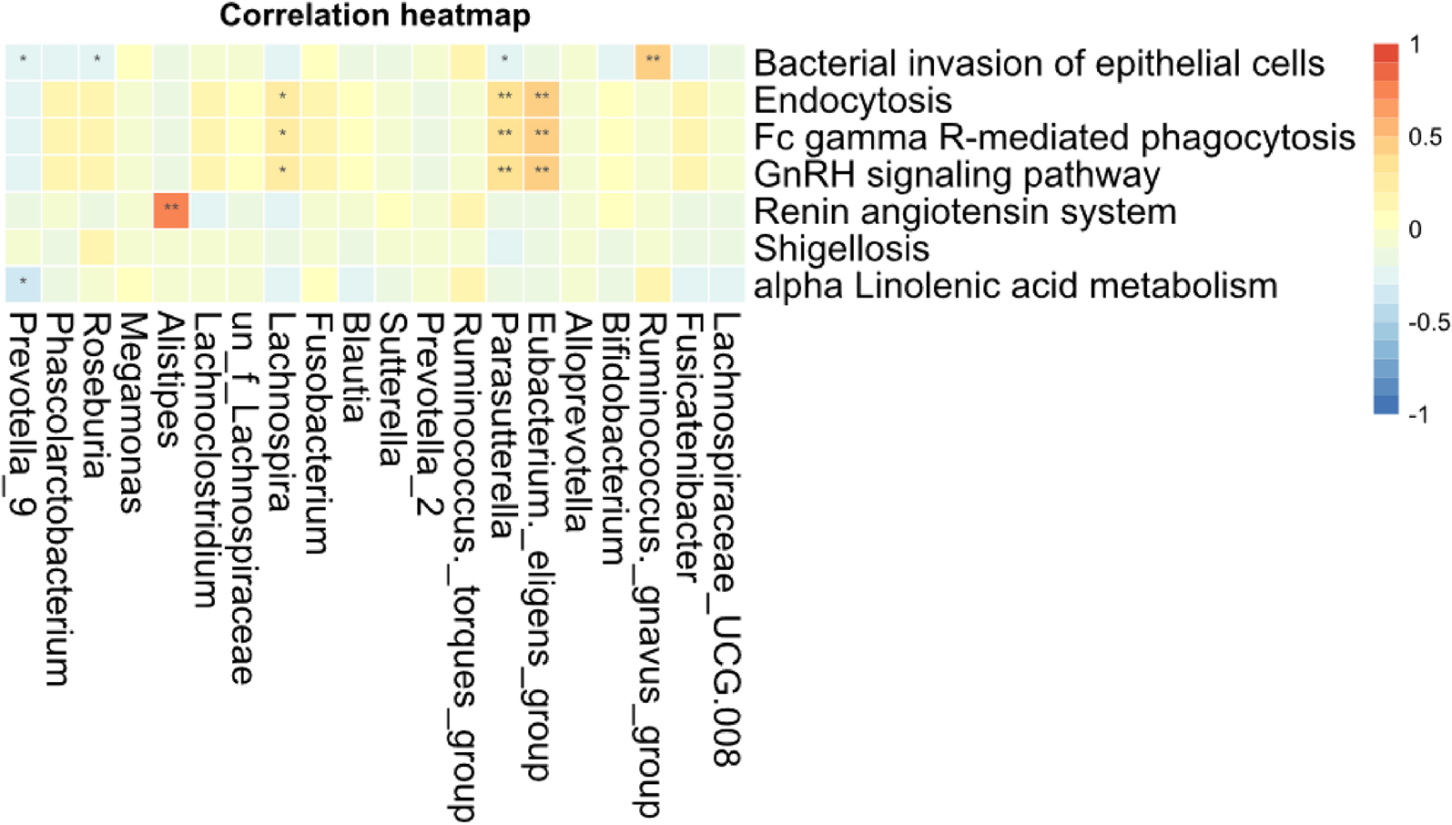
The correlation heatmap between distinguished predicted pathway and bacterial genus. Both differential genus and predicted pathway were screened based on wilcox test (*p* < 0.05) and |logFC|> 1. Correlation significance,* denotes *p* < 0.05 and **p < 0.01

The functional pathway based on 16S rRNA gene were predicted by Picrust to obtain the pathway or metabolites information that may be involved with AR condition. The mean nearest sequenced taxon index (NSTI) value was 0.08 ± 0.02 for all samples (Additional file 1: Table S9). There were 328 detected pathway or metabolites in KEGG level 3 and the top list included transporters, ABC-transporters, purine metabolism, peptidases, pyrimidine metabolism and so on. Then the wilcox test were made to compare the significantly distinguished features and seven of them that were featured with |logFC|> 1 (the expression relative abundance in non-AR group or AR were twice time than AR or non-AR) were screened out (Additional file 1: Table S10). The results showed that the pathway associated with alpha linolenic acid metabolism and bacterial invasion of epithelial cells were significant higher in non-AR group as compare with that of AR, while AR patients showed the higher expression of GnRH signaling pathway, Fc γ-R mediated phagocytosis and Endocytosis (Additional file 2: Figure S3). Furthermore, the correlationship between seven distinguished pathway and the top 20 significantly differed genus were calculated to reveal the potential interaction mode. As is depicted in Fig. 6, no genus significantly related with Shigellosis which were caused by bacterium genus *Shigella*. *Prevotella*_9 negatively related with alpha linolenic acid metabolism. Both *Eubacterium eligens* and *Parasutterella* were negatively correlated with Endocytosis, GnRH signaling pathway and Fc γ-R mediated phagocytosis.

### Moderate and severe AR patients had similar bacterial communities

Based on the TNSS score and actual small sample size, we divided 33 AR patients into moderate (0–7) and severe (8–12) group to compare the bacterial diversity and community to probe into the role of bacterium in the progression of AR. Although moderate and severe AR patients showed significant difference in non-eye/nasal symptoms, practical problem, nasal symptom, eye symptoms, emotions and RQLQ (Additional file 1: Table S11), the α and β diversity displayed no significant difference (Additional file 2: Figure S4), which indicated that at least in this research, bacterial taxa played less roles in the AR progression.

The TNSS score is calculated based on the sum up of nasal obstruction, rhinorrhea, nasal itching, and sneezing, which represents perspectives of AR symptom, that is, the higher score means the worse symptom condition. RQLQ score which consists of activity limitation, sleep disorders, non-eye/nasal symptoms, practical problem, nasal symptom, eye symptoms and emotions, reflects the life quality status that associated with AR disease. The value for each score and RQLQ positively correlated with the extent of the AR impact on the life quality. Here the spearman rank correlation analysis were made to associate TNSS and RQLQ score with bacterial genus. *Butyricicoccus*, *Mailhella*m and *Marvinbryantia* were significantly positive-correlated with TNSS score while opposite for *Filifactor*. *Butyricicoccus*, Lachnospiraceae*_*UCG.001, *Marvinbryantia*, *Prevotella*_2, *Ruminococcaceae_*UCG.013, *Slackia* were the significant genus that positively related with RQLQ while *Eisenbergiella* were negatively associated with RQLQ, eye symptoms, nasal symptom, practical problem, non-eye/nasal symptoms which might need further verification for the therapeutic target of AR disease (Additional file 1: Table S12).

## Discussion

Human gut microbes are key players in host immune responses, metabolism and allergy disease progression [34]. During the study, we compared the composition and functional profiles of gut microbiota between AR patients and their counterparts. The bacterial richness and diversity in AR group was significantly higher than that of non-AR group, which was different from previous findings [35–37]. Most previous studies have shown that the microbial diversity of AR decreased as comparing with non-AR populations [35–37] while few studies found that there was no significant difference in bacterial diversity between AR and non-AR group [38]. According to the sankey diagram of gut microbiota in this study, we found that the higher microbial diversity in AR patients might be due to the expansion of bacteria taxa within Firmicutes.

Generally, opportunistically pathogenic bacteria within healthy gut exerted no problems in immunecompetent hosts while outgrowth of these organisms can contribute to disease [37]. For example, larger proportions of Firmicutes have been reported to be associated with atopic disease, especially in the development of AR and asthma in early childhood [39, 40]. More complicated network topology formed by the expansion of bacterial taxa within Firmicutes in AR group was observed in this study, which indicated the stubborn and leading roles of them in the cross-talk of bacterial community. Hence, adjusting the ecosystem network by modulating the related taxa might be the target for intervention. The proportions of the beneficial microbial organisms were lower in AR group as compared with non-AR group, such as Bacteroidetes and Proteobacteria*,* which have been reported to increase the total numbers of Tregs within the colon. And *B. caccae*, *B. thetaiotaomicron*, and *B. vulgaris* proved to play important roles in protecting host from allergy disease [37]. Also, alterations toward dysbiosis in the composition and predicted function of the gut microbiota in individuals with AR were associated with high total IgE levels [38].

Although the diversity and gut microbiota community between moderate and severe AR group displayed no significant difference, it cannot be absolutely assumed that the gut microbiota plays less role in the AR progression. Many confounding factors can cause disruptions in the structure of the microbial community such as the effect of delivery mode, birth order and family size, antibiotic exposure, diet, breast feeding, indoor or outdoor pollution, occupation, habitation, and socioeconomic factors, etc. [41, 42]. Almost all of the AR individuals included in this study were delivered naturally and breast-fed (Only 2 of 33 cases were caesarean section and 1 of 33 cases was formula fed.). Additionally, individuals in AR group have the similar birth order and family size because of China's one-child policy. Both AR and non-AR individuals live in Xiamen all year round. The lack of differences in gut microbiota between the moderate and severe AR group may be caused by the similar demographic characteristic of AR individuals in this study. Previous studies showed that the maternal vagina and breast milk consisted of *Lactobacilli* and *Streptococci* can reduce the risk of allergy disease as compare with cesarean section and formula fed [43, 44]. Hence, further studies with comparing the impact of confounding factors on AR are needed.

Although there were four primary AR symptoms including nasal congestion, nasal itching, rhinorrhea and sneezing, we found that the clinical manifestations of AR patients varied greatly in real outpatient practice. For example, some AR individuals have serious nasal itching, while other three symptoms were mild; Some AR patients have runny nose, sneezing, but no nasal itching or very mild; Some patients may only have nasal congestion, while other three symptoms were not obvious. We found that the difference of gut microbiota may pose a potential reason for these phenomena through the spearman rank correlation analysis. In this study, *Candidatus_Stoquefichus* was positively related with runny nose; *Catenibacterium* and *Intestinimonas* were positively related with nasal congestion, while opposite for *Alloprevotella*; *Hydrogenoanaerobacterium* and *Prevotella_*6 were positively related with sneezing, while *Granulicatella* and *Porphyromonas* were negatively related with sneezing; *Butyricicoccus, CAG.56,* Family_XIII_UCG.001*, Holdemanella,* and *Hydrogenoanaerobacterium* were positively related with nasal itching, while opposite for *Eisenbergiella* and *Megasphaera.* The above-mentioned results suggest that gut microbiota is positively involved in with the clinical manifestations in varied AR patients.

Regarding to RQLQ, *Butyricicoccus*, Lachnospiraceae*_*UCG.001, *Marvinbryantia, Prevotella_*2, and *Ruminiclostridium* were found to be positively related with activity limitation caused by AR; *Butyricicoccus* and *Slackia* were positively related with non-eye/nasal symptoms, while opposite for *Eisenbergiella*; *Butyricicoccus*, Lachnospiraceae*_*UCG.001, and *Prevotella_*2 were positively related with eye symptoms, while opposite for *Eisenbergiella; Butyricicoccus,* Lachnospiraceae*_*UCG.001, and *Prevotella_*2 were positively related with emotion problem;Lachnospiraceae*_*UCG.001, *Marvinbryantia*, *Prevotella_*2, *Ruminiclostridium* were positively related with practical problem, while opposite for *Eisenbergiella; Marvinbryantia* and *Prevotella_*2 were positively related with nasal symptom, while opposite for *Eisenbergiella; Slackia* was positively related with sleep disorders. Specific probiotics may have huge potential value in the future, which was worthy to be developed to cope with the characteristic symptoms of AR individuals based on these findings.

The potential roles of gut microbiota in AR were predicted in this study. The expression level of predicted function associated with alpha linolenic acid metabolism and bacterial invasion of epithelial cells were higher in non-AR individuals than in AR patients. The bacterial invasion of epithelial cells pathway can destroy the mucosal barrier function which lead to the intrusion of exogenous pathogens. It was well known that the gut microbiota played a key role in the formation of mucosal immune system. In terms of function, the gut mucosa and nasal mucosa may seem as a single organ, which sharing the functions of immune surveillance as well as shaping of host responses [45, 46]. The balance between T type 17 cells and T regulatory cells in the lamina propria of the small intestine can be regulated by the gut microbiota [45]. The symbiotic relationship between intestinal mucosa and the gut microbiome has a barrier mechanism to exclude most bacteria, so as to prevent undesired invasion and the tolerance will be developed through continuous exposure to bacterial products. In AR patients, pathobionts accounted for a large proportion which can invasion the intestinal mucosa epithelial cells leading to barrier function dysbiosis. The dendritic cells and macrophages can be affected by the pathobionts and metabolic by-products directly or through the intermediacy of epithelial cells, which is regulated by microbiota-driven epigenetic mechanisms. Besides, the microbial metabolic products also can induce the maturation of T regulatory cells as well as A and B cell, causing such cells to switch their immunoglobulin isotypes. For example, IgE modifies the gut microbiota via activating basophils and mast cells. The development of gut mucosal immunity which can prevent the intrusion of exogenous pathogens is stimulated by the cross-talk between the gut microbiota and the immune system [42].

There were several limitations to this study. Firstly, we included limited number of subjects in each group with a cross-sectional design confined to Xiamen area, which remind of us that more samples across different geographic area should be considered to make more robust conclusion. Secondly, although we know diet, lift style and many other confounding factors induce gut microbiota dysbiosis, the effect of environmental factors, including indoor, outdoor pollution, occupation, habitation and socioeconomic factors were not included in the analysis [47]. Thirdly, biomarkers of respiratory inflammation such as peripheral blood eosinophil, nasal mucus eosinophil or IgE were not fully assessed in this study. Fourth, although 16S rRNA PICRUSt analysis has been adopted in this human gut microbiota research to help us further comprehend the potential function and pathway related with allergy rhinitis, the less accurate results associated the inherent drawback of database and tools should be further improved by metagenome, transcriptome and other omics tools. In addition, although we collected the nasal mucosa flora, the quantity of the nasal mucosa flora was too small to carry out 16S rRNA sequencing that we could not know the potential influence of upper airway microbiota on AR. AR seems to be a self-healing disease with the growing of age and increasing of immunity for some AR children. But clinically, we still see a lot of AR patients, who suffering from AR from children to adults and even to the elderly. Unfortunately, this study did not include the children or the elderly AR patients, so we do not know which certain microbiota plays a key role in the progress of AR. Hence, further studies of larger sample size, multi-omics, host-microbiome interaction are needed to validate these findings, which will guide us the precise roles of gut microbiota in the pathogenesis and progression of AR.

## Conclusion

Both the bacterial α and β diversity were significantly different between AR and non-AR individuals. The potentially beneficial bacterial related to reduce nasal symptoms and improve the patients’ quality of life were lower in AR patients, whereas taxa associated with enhancement of nasal symptoms and decrease of the patients’ quality of life were higher in AR patients. These results suggest that the alteration of gut microbiota can be associated with AR through their functional roles. This study sets the basis for gut microbiota regulation as a potential therapeutic target for allergy rhinitis.

## Supplementary information

**Additional file 1:** **Table S1. **Basic clinical information of included patients and healthy individuals. **Table S2.** Sequence process information about AR patients and non-AR healthy individuals. **Table S3. **Bacterial diversity among AR patients and non-AR healthy individuals**. Table S4. **Bacterial taxa abundance in phyla level. **Table S5. **Characteristic bacterial biomarkers screened by LEfSe. **Table S6. **Bacterial taxa abundance in genus level. **Table S7. **Top 20 distinguished genus between AR patients and non-AR healthy individuals. **Table S8. **The nodes and edge information of non-AR healthy and AR group. **Table S9. **The NSTI values in each sample indicate the accuracy of the predicted metabolic potential. **Table S10. **The distinguished predicted pathway between AR patients and non-AR healthy individuals. **Table S11.** Baseline comparison between mild-to-moderate and severe AR patients. **Table S12. **The Spearman rank correlation between bacterial genus, TNSS and quality of life score.

**Additional file 2:** **Figure S1.** The rarefaction curves of all sample. (A) Observed species and (B) chao1. **Figure S2.** The bacterial community difference between AR and non-AR group. (A) The ANOSIM analysis are made to compare the intergroup and within group of samples. (B) The bacterial community variation comparison in AR and non-AR group. **Figure S3.** The predicted expression of distinguished pathway. The significantly distinguished pathway predicted by PICRUSt were screened by comparison between AR and non-AR individuals by Wilcox test and based on |logFC|>1. FC denotes fold change. **Figure S4.** Comparisons of bacterial diversity between moderate and severe AR patients. (A) The bacterial α diversity indexes comparison between moderate and severe AR, including Chao1, J and Shannon. Letters indicate the ANOVA groupings. (B) Differences in bacterial community structures between moderate and severe AR patients.

## Data Availability

The dataset supporting the conclusions of this article is available in the NCBI Sequence Read Archive repository under the accession number PRJNA624106 (https://submit.ncbi.nlm.nih.gov/subs/). Code and scripts used in the analyses are available upon request.
