# Correction to: Gut microbial characteristics of adult patients with allergy rhinitis

**DOI:** 10.1186/s12934-020-01441-x

**Published:** 2020-10-08

**Authors:** Libing Zhu, Feng Xu, Wenrong Wan, Bin Yu, Lin Tang, Yiming Yang, Yanling Du, Zhangran Chen, Hongzhi Xu

**Affiliations:** 1 Department of Traditional Chinese Medicine, School of Medicine, Xiamen University, Xiamen, China; 2 Internal Medicine Department of Traditional Chinese Medicine, Xiamen Hospital of Traditional Chinese Medicine, Xiamen, China; 3 Department of Otorhinolaryngology, Xiamen Hospital of Traditional Chinese Medicine, Xiamen, China; 4 Department of Pediatrics, Xiamen Hospital of Traditional Chinese Medicine, Xiamen, China; 5 Department of Acupuncture and Tuina, Fujian University of Traditional Chinese Medicine, Fuzhou, China; 6 Department of Digestive Diseases, School of Medicine, Xiamen University, Xiamen, China; 7 Department of Gastroenterology, Zhongshan Hospital, Xiamen University, Xiamen, China; 8 Institute for Microbial Ecology, School of Medicine, Xiamen University, Xiamen, China

## Correction to: Microb Cell Fact (2020) 19:171 https://doi.org/10.1186/s12934-020-01430-0

Following publication of the original article [1], the authors identified two errors: one in the author name of Hongzhi Xu, and one in the caption of Fig. 1.

The author name Hongzhi Xu had been (incorrectly) spelled as ‘Hongzhu Xu’.

While Fig. 1 had been (incorrectly) captioned with ‘Comparisons of bacterial diversity between AR and non-AR patients. a The bacterial á diversity indexes comparison including Chao1, J and Shannon. Letters indicate the ANOVA groupings. b Differences in bacterial community structures between samples from AR and non-AR’

**Fig. 1:**
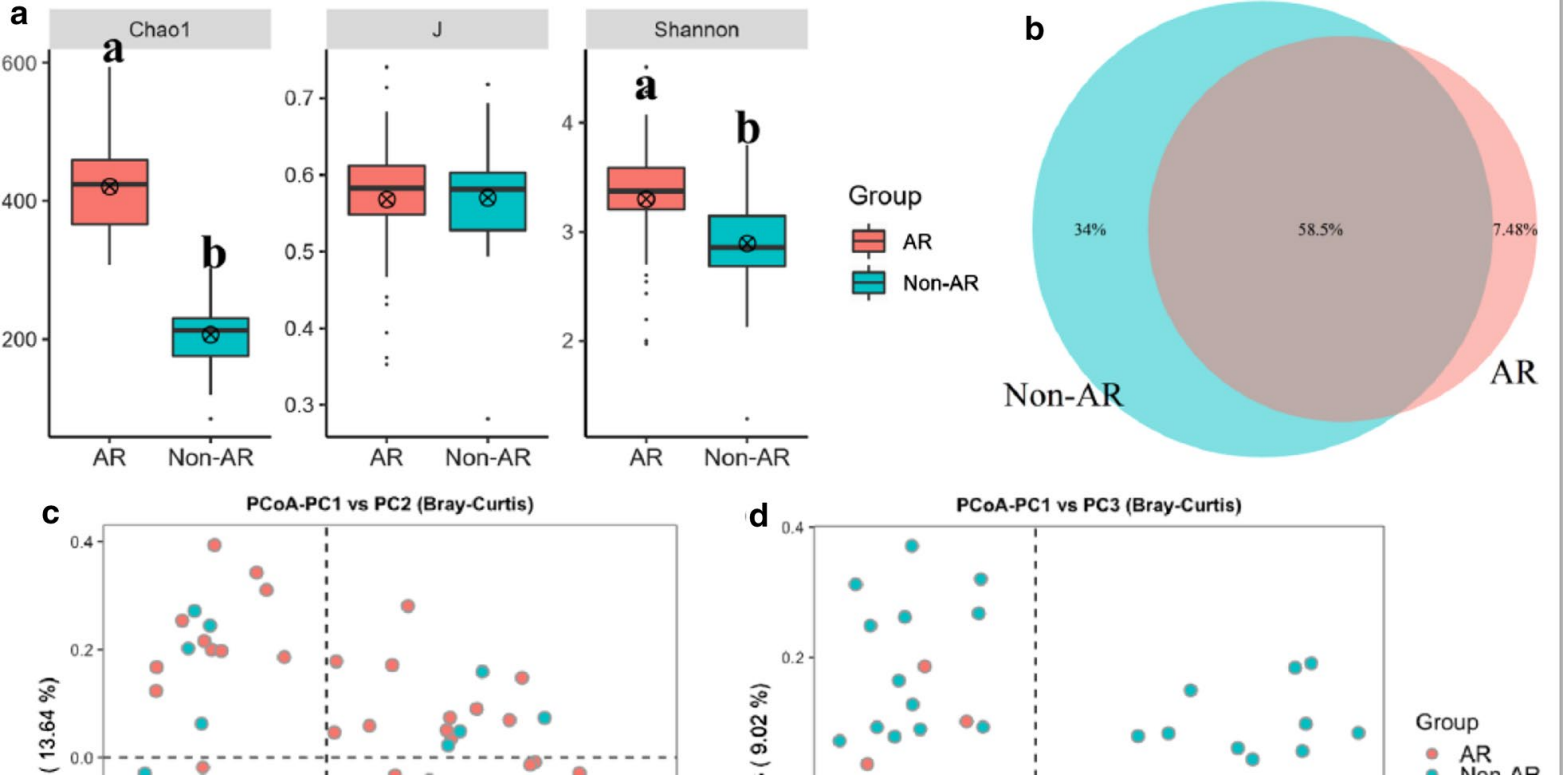
The PCoA analysis revealing the bacterial communities between AR and non-AR in PCoA1 vs PCoA 2 axis (**c**) and PCoA1 vs PCoA 3 axis (**d**).

The name and caption have been corrected in the original article.

Please also find the corrected author name and figure caption in this correction article.

The authors apologize for any inconvenience caused.
