# Supplementary material for: Gut microbial characteristics of adult patients with allergy rhinitis
Source: Microb Cell Fact. 2020 Sep 1;19:171. doi: 10.1186/s12934-020-01430-0 (PMC7466420; doi:10.1186/s12934-020-01430-0)
Supplement: Supplementary file 2 — Additional file 2: Figure S1. The rarefaction curves of all sample. (A) Observed species and (B) chao1. Figure S2. The bacterial community difference between AR and non-AR group. (A) The ANOSIM analysis are made to compare the intergroup and within group of samples. (B) The bacterial community variation comparison in AR and non-AR group. Figure S3. The predicted expression of distinguished pathway. The significantly distinguished pathway predicted by PICRUSt were screened by comparison between AR and non-AR individuals by Wilcox test and based on |logFC|>1. FC denotes fold change. Figure S4. Comparisons of bacterial diversity between moderate and severe AR patients. (A) The bacterial α diversity indexes comparison between moderate and severe AR, including Chao1, J and Shannon. Letters indicate the ANOVA groupings. (B) Differences in bacterial community structures between moderate and severe AR patients. [file 12934_2020_1430_MOESM2_ESM.docx]

**Figure S1.** The rarefaction curves of all sample. (A) Observed species and (B) chao1.

**(A)**


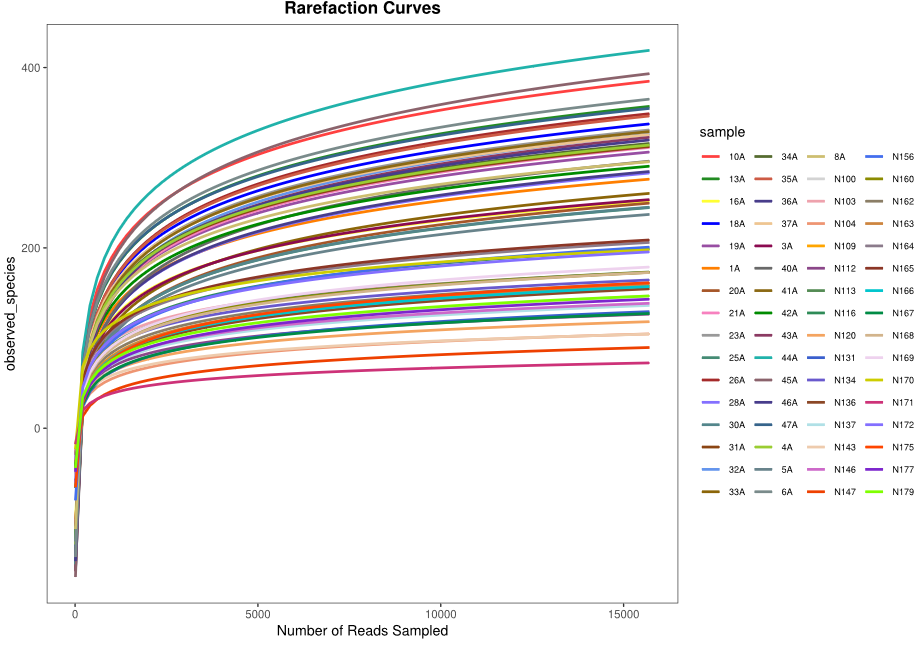


**(B)**


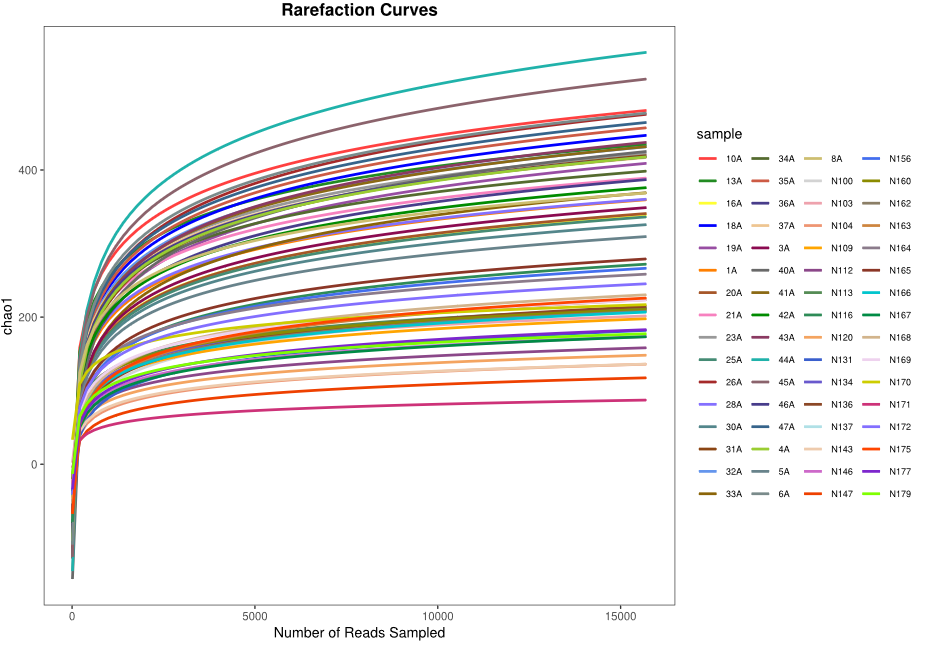


**Figure S2.** The bacterial community difference between AR and non-AR group. (A) The ANOSIM analysis are made to compare the intergroup and within group of samples. (B) The bacterial community variation comparison in AR and non-AR group.


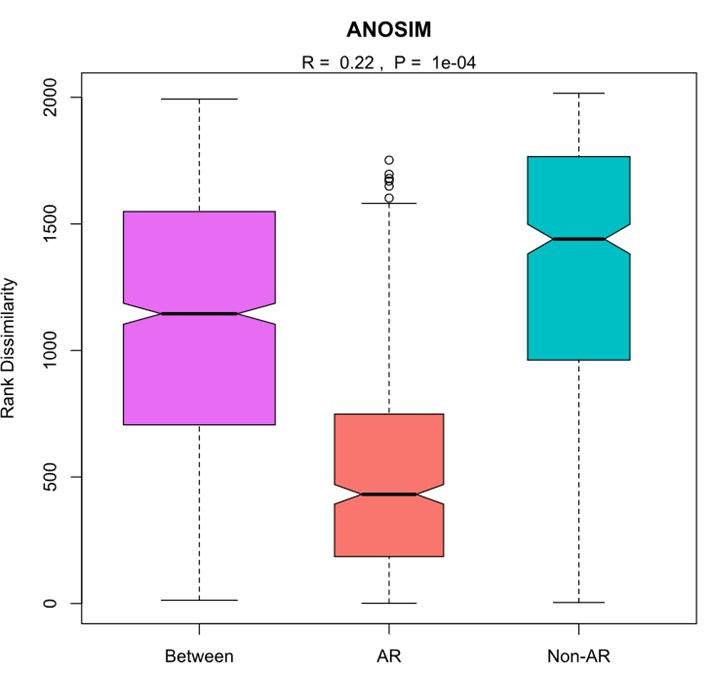


**(A)**

**(B)**


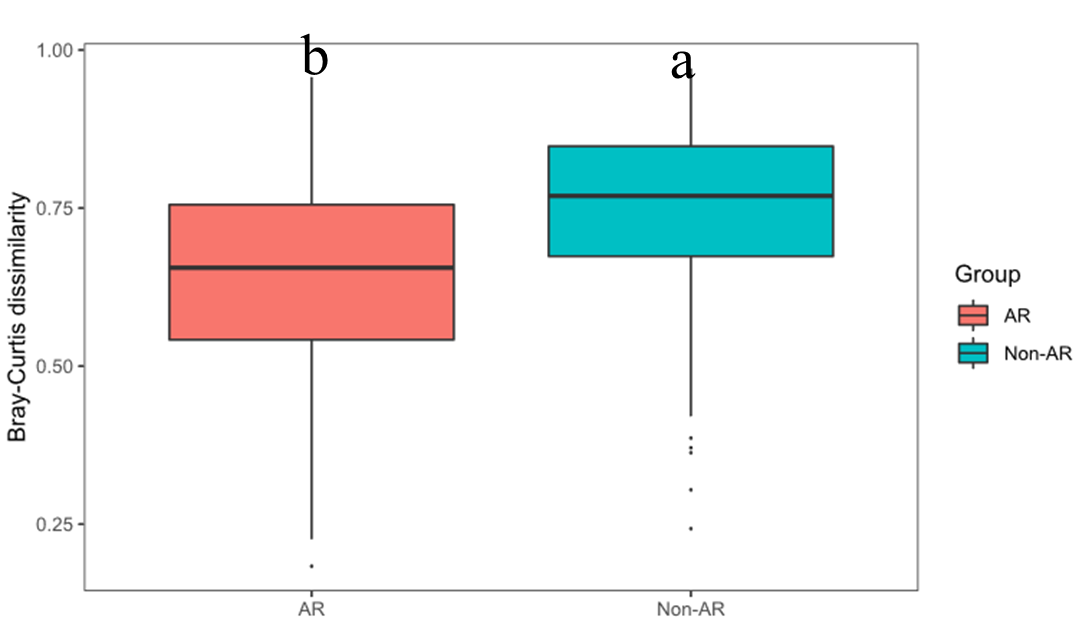


**Figure S3.** The predicted expression of distinguished pathway. The significantly distinguished pathway predicted by PICRUSt were screened by comparison between AR and non-AR individuals by Wilcox test and based on |logFC|>1. FC denotes fold change.


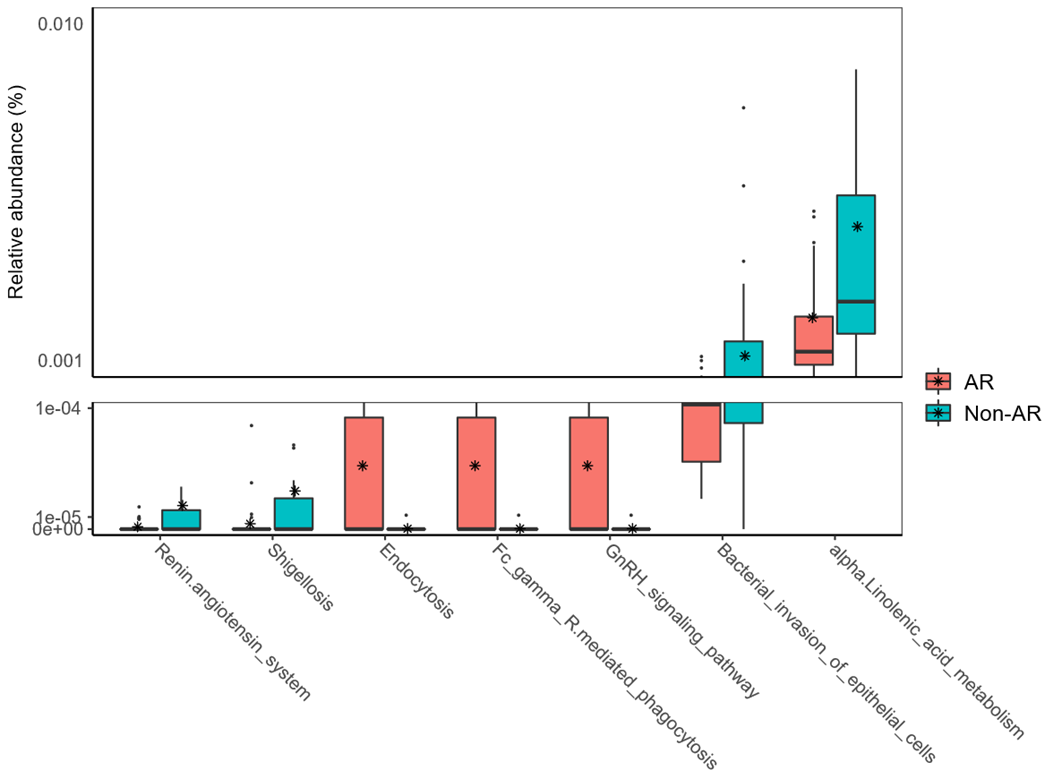


**Figure S4.** Comparisons of bacterial diversity between moderate and severe AR patients. (A) The bacterial α diversity indexes comparison between moderate and severe AR, including Chao1, J and Shannon. Letters indicate the ANOVA groupings. (B) Differences in bacterial community structures between moderate and severe AR patients.

**(A)**


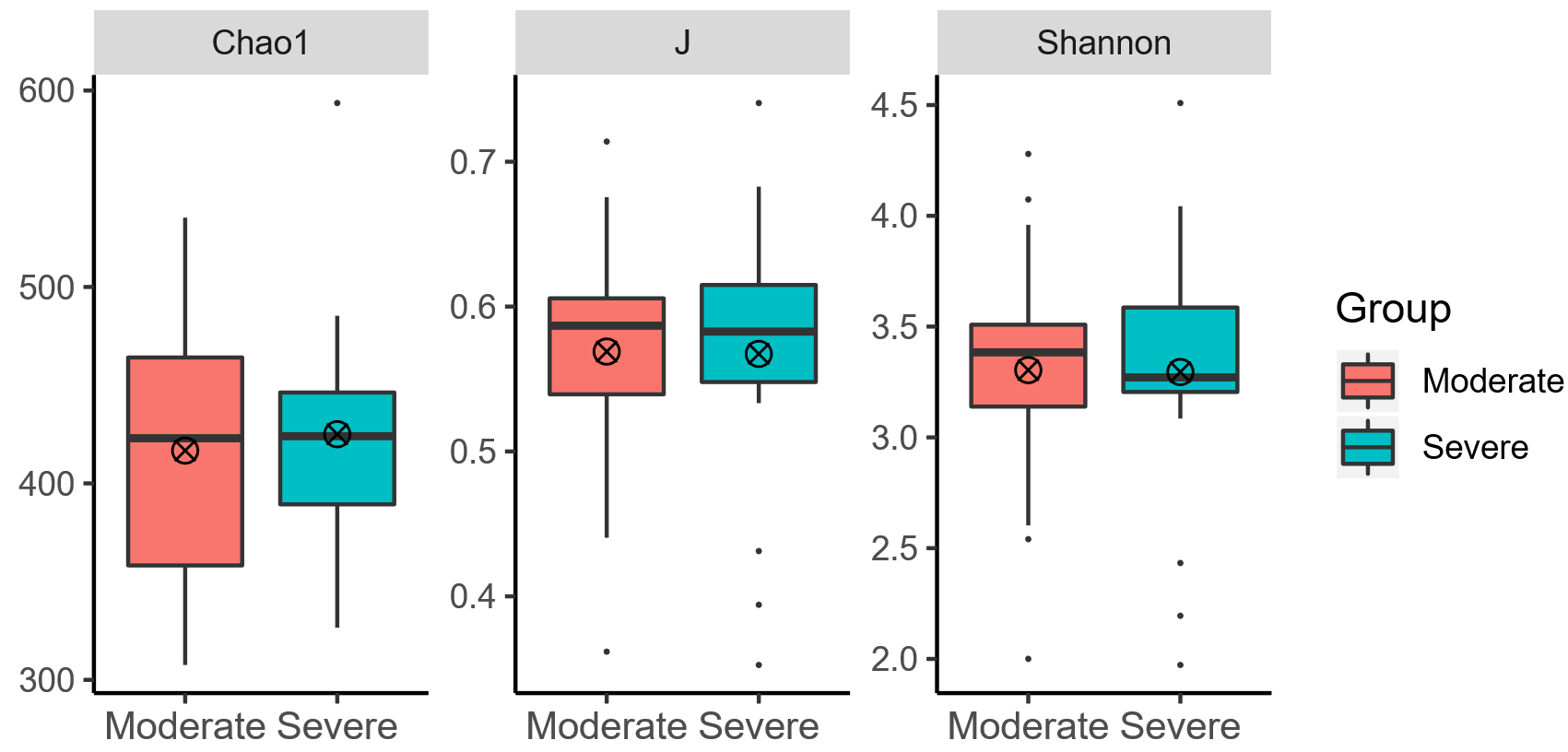

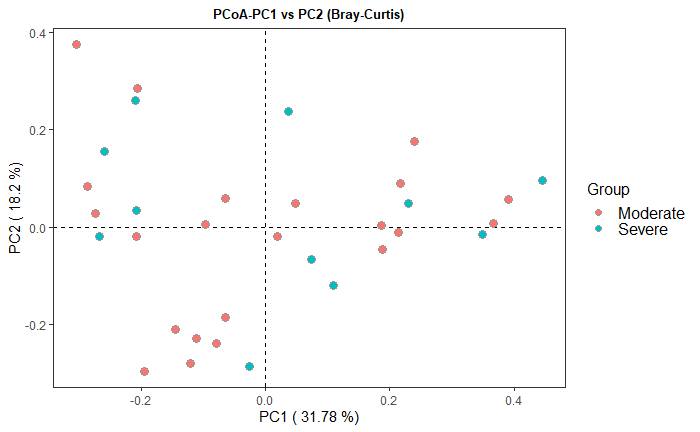


**(B)**
